# Targeting mTORC2 inhibits colon cancer cell proliferation in vitro and tumor formation in vivo

**DOI:** 10.1186/1476-4598-9-57

**Published:** 2010-03-12

**Authors:** Didier Roulin, Yannick Cerantola, Anne Dormond-Meuwly, Nicolas Demartines, Olivier Dormond

**Affiliations:** 1 Department of Visceral Surgery, Centre Hospitalier Universitaire Vaudois and University of Lausanne, Pavillon 3, Av de Beaumont, 1011 Lausanne, Switzerland

## Abstract

The mammalian target of rapamycin (mTOR), which exists in two functionally distinct complexes, mTORC1 and mTORC2 plays an important role in tumor growth. Whereas the role of mTORC1 has been well characterized in this process, little is known about the functions of mTORC2 in cancer progression. In this study, we explored the specific role of mTORC2 in colon cancer using a short hairpin RNA expression system to silence the mTORC2-associated protein rictor. We found that downregulation of rictor in HT29 and LS174T colon cancer cells significantly reduced cell proliferation. Knockdown of rictor also resulted in a G1 arrest as observed by cell cycle analysis. We further observed that LS174T cells deficient for rictor failed to form tumors in a nude mice xenograft model. Taken together, these results show that the inhibition of mTORC2 reduces colon cancer cell proliferation in vitro and tumor xenograft formation in vivo. They also suggest that specifically targeting mTORC2 may provide a novel treatment strategy for colorectal cancer.

## Findings

The mammalian target of rapamycin (mTOR) is a serine-threonine kinase that regulates cell growth and proliferation in response to the availability of growth factors and nutrients [1,2]. mTOR exists in two functionally distinct complexes: mTORC1 and mTORC2, which have different functions and are differentially sensitive to rapamycin [3]. mTORC1 is composed of mTOR, mLST8, PRAS40, deptor and raptor and is sensitive to rapamycin. mTORC1 enhances cell growth and proliferation through various mechanisms including synthesis of proteins and lipids as well as reduction of autophagy. Part of the functions of mTORC1 are mediated by the phosphorylation and activation of S6K1 and 4E-BP1, two well characterized downstream effectors of mTORC1 [1]. mTORC2 contains mTOR, mLST8, mSIN1, deptor, protor-1 and rictor. Although mTORC2 is classically insensitive to rapamycin, prolonged exposure to rapamycin may also inhibit mTORC2 activity by blocking its assembly in certain cell types [4]. In contrast to mTORC1, relatively little is known about the functions of mTORC2. It has been described that mTORC2 regulates cell survival and cytoskeletal organization through the regulation of Akt and PKCα respectively [5,6].

Since mTOR plays a major role in cell growth and proliferation, targeting mTOR in cancer therapy has been viewed as a promising approach [3,7]. Indeed, alterations of mTOR signaling pathway are commonly observed in solid tumors. Whereas the role of mTORC1 in cancer progression has been extensively characterized, the role of mTORC2 is much less documented. So far it has been shown that targeting mTORC2 might be beneficial in tumors harboring high levels of activated Akt, such as gliomas or tumors caused by PTEN deletion [8,9]. Since the activation of Akt is frequently observed in colorectal cancer [10], we wish here to analyse the role of mTORC2 in colon cancer.

**Figure 1:**
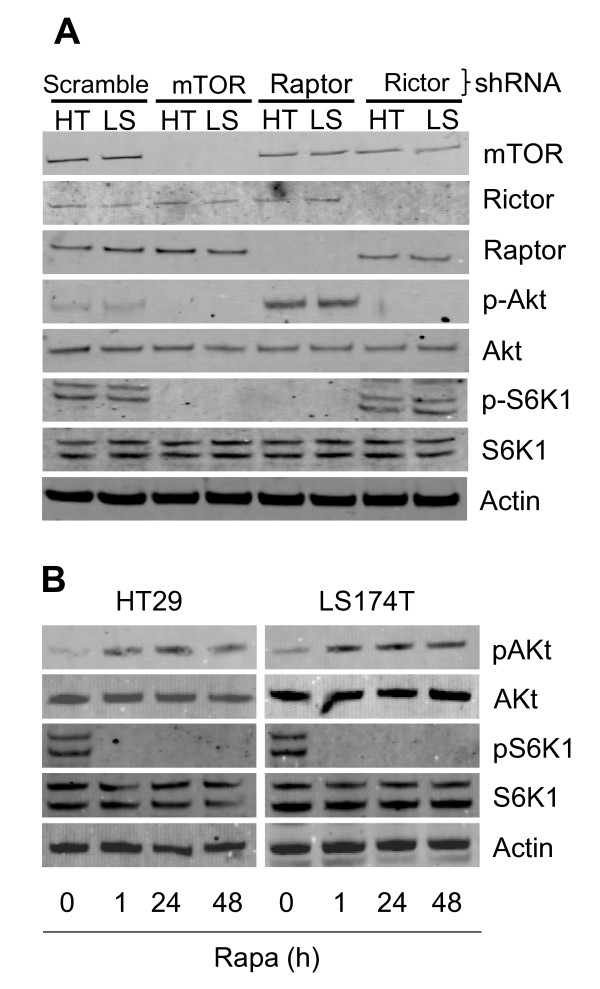
**Downregulation of mTOR, rictor and raptor in HT29 and LS174T colon cancer cells**. (A) Knockdown of raptor, rictor or mTOR in HT29 (HT) and LS174T (LS). HT29 and LS174T cells were infected with recombinant lentiviruses containing the indicated shRNA. Two days post infection cells were selected for resistance to puromycin for 48 hours. Resistant cells were plated in 6 well plates and cultured in DMEM 10% FBS for 2 days. Cells were subsequently lysed in RIPA lysis buffer containing a protease inhibitor cocktail (Sigma) and 1 mM sodium orthovanadate. Proteins were quantified with the BCA protein Assay Kit (Pierce). Equal amounts of proteins (30 μg) were resolved on SDS PAGE, transferred to membranes and immunoblotted with primary antibodies against mTOR, rictor, raptor, phospho-Akt (Ser 473), Akt, phospho-S6K1 (Thr 389) or S6K1 (Cell Signalling Technology) followed by infrared secondary antibodies (LI-COR Biosciences). Bands from immunoreactive proteins were visualised by an Odyssey infrared imaging system (LI-COR Biosciences). (B) Rapamycin inhibits mTORC1 but not mTORC2 in HT29 and LS174T cells. HT29 or LS174T cells were treated with rapamycin (10 ng/ml) for the indicated times. Cells were subsequently lysed and Western Blot analysis was performed as in A. The illustrated blots are representative of three with similar findings.

To investigate the role of mTORC2 in colon cancer, we used a lentiviral short hairpin RNA (shRNA) expression system that suppresses the expression of rictor to block the activity of mTORC2 [5]. In addition we also suppressed the expression of raptor to block mTORC1 or the expression of mTOR to block both complexes. HT29 and LS174T colon cancer cells were infected with lentiviruses expressing either a scramble shRNA, raptor shRNA, rictor shRNA or mTOR shRNA. The gene knockdown efficiency following infection was analysed by Western Blot. Using this technique, we observed that raptor, rictor and mTOR expression was significantly reduced in both HT29 and LS174T cells (Figure 1A). We further observed that mTOR and raptor downregulation reduced the phosphorylation of S6K1, a downstream target of mTORC1. In addition, we also found that mTOR and rictor shRNA significantly reduced the phosphorylation of Akt, a downstream effector of mTORC2. Finally, targeting raptor increased the phosphorylation of Akt consistent with a previously reported negative feedback loop whereby inhibition of mTORC1 induces the activation of Akt (Figure 1A) [3]. These results show that mTORC1 and mTORC2 activity can be efficiently blocked by raptor or rictor shRNA respectively in HT29 and LS174T cells. To further document the role of mTOR in colon cancer cells, we also treated HT29 and LS174T cells with rapamycin, a chemical inhibitor of mTORC1. Similarly to the inhibition of mTORC1 by raptor shRNA, we found that, while blocking S6K1 phosphorylation, rapamycin increased Akt phosphorylation, These results show that rapamycin inhibits mTORC1 but not mTORC2 in HT29 and LS174T cells (Figure 1B).

**Figure 2:**
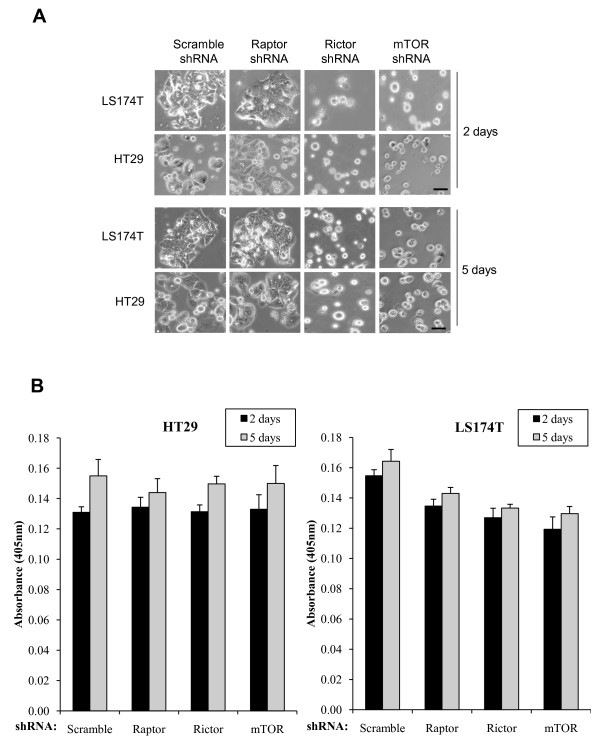
**Morphological changes induced by mTORC2 knockdown in colon cancer cells**. (A) Morphological effects of mTOR and Rictor downregulation in HT29 and LS174T cells. HT29 or LS174T cells were infected with lentiviruses expressing shRNAs targeting the indicated genes. Following selection with puromycin for 48 hours, cells were grown on a 6 well plate for 2 or 5 additional days, and pictures were taken under a phase-contrast microscope at × 100 magnification. Scale bar: 50 μm. (B) Effects of mTOR, rictor or raptor downregulation on HT29 and LS174T cell apoptosis. HT29 and LS174T cells were processed as in A. After 2 or 5 days of culture, equal amount of cells were harvested and apoptosis was measured by quantifying DNA fragmentation using the Cell Death Detection Elisa Plus (Roche) as described by the manufacturer. Results are represented as the mean absorbance at 405 nm ± standard deviations (n = 3).

After selection by puromycin for 48 hours, we also found that knockdown of mTOR and rictor significantly changed the morphology of HT29 and LS174T cells. While HT29 and LS174T cells had a small distinct cytoplasm and grew in cell clusters, mTOR or rictor deficient HT29 and LS174T cells were small and round with absent cytoplasm, and which failed to grow (Figure 2A). In contrast, raptor knockdown did not alter the morphology of HT29 and LS174T cells. To exclude that the morphological changes observed in mTOR and rictor knockdown cells was indeed due to an increased cell death, we monitored apoptosis by quantifying DNA fragmentation. We did not find a significant change in cell apoptosis between cells expressing scramble, raptor, mTOR or rictor shRNA (Figure 2B).

**Figure 3:**
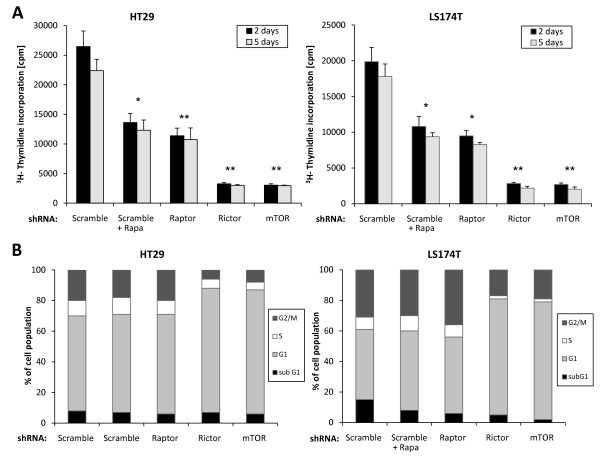
**Effects of mTORC2 knockdown on HT29 and LS174T cell proliferation**. (A) Knockdown of mTOR or rictor reduces the proliferation of HT29 and LS174T cells. HT29 and LS174T cells were infected with lentiviral particles containing scramble, raptor, rictor or mTOR shRNA. Forty-eight hours post-infection cells were selected for resistance to puromycin for an additional 48 hours. Subsequently, 5 × 10^3 ^cells were plated on 96 well plates and cultured in DMEM 10% FBS for 2 or 5 days. Cell proliferation was determined by ^3^H-thymidine incorporation in the final 12 hours of cell culture. Results are expressed as the mean cpm ± standard deviation of three experiments. *p < 0.05 and **p < 0.01 compared with cells expressing the scramble shRNA (Student's *t *test). (B) Knockdown of mTOR or rictor induces a G1 arrest in HT29 and LS174T cells. HT29 and LS174T cells were processed as in A. Following selection for resistance to puromycin cells were cultures for 2 days and subsequently collected by trypsin digestion, washed in PBS incubated for 24 hours in 70% ethanol. Cells were subsequently resuspended in PBS containing 20 μg/ml propidium iodide and 200 μg/ml RNAse and incubated for 45 minutes at 37°C. DNA content of the cells was analysed with a FACScan II and the CellQUEST software (Beckton Dickinson). One of three similar experiments is shown.

To further characterize the role of mTORC2 in colon cancer progression, we analyzed the effect of rictor knockdown on colon cancer cell proliferation. We found that the proliferation of HT29 and LS174T cells deficient for rictor or mTOR was markedly reduced (Figure 3A). Knockdown of raptor also significantly reduced the proliferation of HT29 and LS174T cells compared to control cells, however to a lesser extend than rictor or mTOR knockdown. Finally, rapamycin treatment also reduced cell proliferation to a similar extend than raptor knockdown. To gain insight into the mechanism by which rictor and mTOR knockdown reduced colon cancer cell proliferation, we analyzed cell cycle progression using propidium iodide staining and flow cytometry analysis. We found that mTOR or rictor knockdown in LS174T cells resulted in a G1 arrest as observed by a marked increase in the G1 population. Similar results were observed in HT29 cells (Figure 3B).

**Figure 4:**
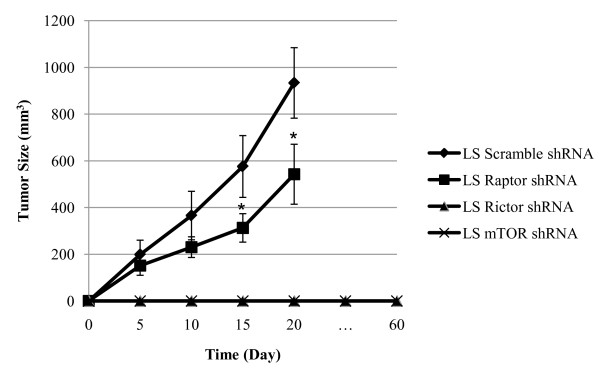
**Colon cancer cells require mTOR or rictor to form tumors as xenografts**. LS174T cells were infected with lentiviral particles containing scramble, raptor, rictor or mTOR shRNA. Stable transfectants were selected for resistance to puromycin for 48 hours and subsequently cultured in DMEM 10% FBS for 2 days. An equal amount (1 × 10^6^) of LS174T cells deficient for raptor, rictor, or mTOR, or expressing a scramble shRNA were harvested and injected subcutaneously into immunodeficient mice (n = 5 in each group). Tumor volumes were evaluated using calliper measurements and calculated with the formula V = π/6 × *a*^2 ^× *b *where *a *is the short axis and *b *the long axis of the tumor. Mice bearing the scramble or the raptor shRNA xenografts were sacrificed after 20 days. Mice bearing the mTOR or the rictor shRNA xenografts were observed during 60 days. *Points*, average value of tumor volume. *Bars*, SD. Animal experiments were in accordance with the Swiss federal animal regulations and approved by the local veterinary office. *p < 0.001 comparing with cells expressing the Scramble shRNA (one-way ANOVA).

We next tested the effect of mTOR and rictor knockdown on tumor growth in vivo. LS174T cells deficient for mTOR, raptor and rictor as well as LS174T expressing a scramble shRNA as a control were injected subcutaneously into nude mice and tumor growth was monitored. We found that mTOR or rictor deficient LS174T cells failed to form a tumor xenograft even after 60 days of observations (Figure 4). In contrast, LS174T cells deficient for raptor formed tumor xenografts which grew however slower than control xenografts.

Several studies have demonstrated that the activity of mTOR is increased in tumors [7]. Particularly in colorectal cancer, mTOR signaling components were highly activated in tumor specimen compared to non cancerous mucosa [11]. Furthermore, targeting mTOR with a specific siRNA to mTOR also reduced SW480 and HCT116 colon cancer cell proliferation and survival in vitro and injection of small interfering RNA to mTOR into HCT116 tumor xenografts also blocked tumor growth in vivo [11]. In addition, the inhibition of mTORC1 suppressed the formation of colonic adenomas and cancers in a mouse model of familial adenomatous polyposis [12]. Therefore, targeting mTOR in colorectal cancer might be a successful strategy. mTOR exists in two distinct complexes, however, no study so far has analyzed the specific role of mTORC2 in colorectal cancer development. Here, we found that mTORC2 plays an important role in colon cancer cell proliferation. Downregulation of mTORC2, by either blocking the expression of mTOR or rictor, reduced the proliferation of HT29 and LS174T colon cancer cells in vitro and inhibited the formation of tumor xenografts in vivo. We therefore propose that targeting mTORC2 in colon cancer could be a promising therapeutical strategy.

Targeting mTOR with rapamycin or its analogs in clinical studies has been less successful than expected [7]. However, as mTORC2 and part of mTORC1 functionality are resistant to rapamycin, one may speculate that therapies that target both complexes will be much more efficient in cancer therapy. Consistent with this hypothesis, we found that the inhibition of mTORC1 reduced colon cancer cell proliferation, however to a lesser extend than the inhibition of both complexes, or mTORC2 alone. Recently, several groups have developed ATP-competitive and selective mTOR inhibitors that target simultaneously both complexes [13-15]. Initial experiments have shown that the antiproliferative efficacy of these inhibitors is superior to rapamycin [15]. Future studies will reveal the efficacy of such inhibitors in cancer therapy; one major concern being that these inhibitors might have considerable toxicity in vivo. However, our results suggest that targeting only mTORC2 might be sufficient to prevent colon cancer progression and thus give a rationale for the development of drugs that specifically target mTORC2.

## Abbreviations

mTOR: mammalian target of rapamycin; shRNA: small hairpin RNA.

## Competing interests

The authors declare that they have no competing interests.

## Authors' contributions

DR, YC, ADM performed the experiments and interpreted the experimental findings. YC and OD conceived the study. DR drafted the manuscript. ND and OD wrote the final version of the manuscript. All authors read and approved the final manuscript.
